# Human Illnesses Caused by *Opisthorchis felineus* Flukes, Italy

**DOI:** 10.3201/eid1412.080782

**Published:** 2008-12

**Authors:** Orlando Armignacco, Luciano Caterini, Gianluca Marucci, Fabrizio Ferri, Giuliana Bernardini, Giampaolo Natalini Raponi, Alessandra Ludovisi, Teresa Bossù, Maria A. Gomez Morales, Edoardo Pozio

**Affiliations:** Belcolle Hospital, Viterbo, Italy (O. Armignacco, L. Caterini, F. Ferri, G. Bernardini); Istituto Superiore di Sanità, Rome, Italy (G. Marucci, A. Ludovisi, M.A. Gomez Morales, E. Pozio); S. Camillo de Lellis Hospital, Rieti, Italy (G. Natalini Raponi); Istituto Zooprofilattico Sperimentale of Latium and Tuscany, Rome (T. Bossù)

**Keywords:** Opisthorchiasis, eosinophilia, transaminases, Opisthorchis felineus, zoonosis, outbreak, freshwater fish, Tinca tinca, Italy, dispatch

## Abstract

We report 2 outbreaks of *Opisthorchis felineus* infection caused by the consumption of tench filets (*Tinca tinca*) from a lake in Italy. Of the 22 infected persons, 10 (45.4%) were asymptomatic. When present, symptoms (fever, nausea, abdominal pain, and myalgias) were mild. Eosinophilia occurred in all infected persons.

*Opisthorchis felineus* is a trematode that is transmitted to humans through the consumption of raw freshwater fish of the family Cyprinidae. Worldwide, the number of cases of human infection has been estimated to be 1.2 million (*1*). A high prevalence has been reported in Byelorussia, Russia, and the Ukraine. In the European Union, sporadic human infections have been documented in Germany, where the parasite has been detected in red foxes and cats, and in Greece (*2**–**6*).

In Italy, *O. felineus* was first described in cats and dogs in Pisa (Tuscany Region) and in cats in Turin (Piedmont Region), yet for over 100 years the infection was not detected or reported in animals and humans and no one investigated this pathogen (*7**,**8*). With regard to human infection, cases were reported in 2003 and 2005, when 2 outbreaks of opisthorchiasis occurred after persons consumed fish from Lake Trasimeno (central Italy) (*9*). Our study describes 2 recent outbreaks and provides the results of the epidemiologic investigation.

## The Study

In August 2007, an outbreak in central Italy involved persons who had consumed fish at a private dinner. In October–November 2007, a second outbreak involved persons who had also eaten fish. For both outbreaks, index case-patients were interviewed to trace others who had eaten these meals. A case of opisthorchiasis was defined as *Opistorchidae* eggs in a fecal sample or immunoglobulin (Ig) G antibodies to *Opisthorchis* spp. in a serum sample from persons who had consumed raw freshwater fish.

We searched for parasites in fecal samples after formol-ether concentration by light microscopy. To investigate the presence of trematodes in fish from the lake where they had been caught, 800 specimens of 17 species were collected. Muscle tissues from these fish were digested with 1% pepsin and 1% HCl at 40°C to detect metacercariae.

Stool samples (4 g) were concentrated by a modified formalin-ethyl acetate procedure. Parasite DNA was purified from 200 µL of fecal pellets by using the QIAamp DNA stool kit (QIAGEN, Hilden, Germany), following the manufacturer’s instructions. The primers OP1 (5′-CGAGGGTCGGCTTATAAAC-3′) and OP2 (5′-AGCCTCAACCAAAGACAAAG-3′) were used to amplify the ITS2 region of the rDNA of the parasite eggs and metacercariae (*10*). The 250-bp fragment was sequenced and compared with the internal transcribed spacer (ITS2) sequences of *O. felineus*, *O*. *viverrini*, and *Clonorchis sinensis* present in the GenBank database. We used ELISA to search for IgG antibodies to *O. felineus* in blood samples by using excretory/secretory antigens, according to a standard protocol (*11*).

**Figure 1:**
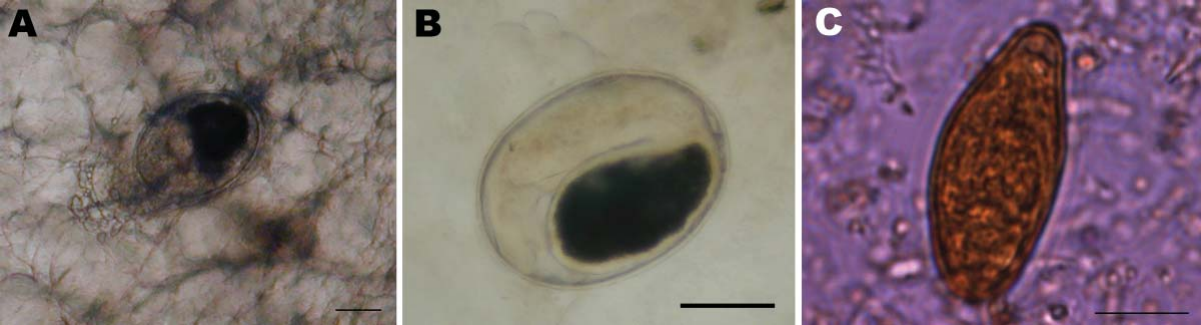
A) Metacercaria of *Opisthorchis felineus* in muscles of a tench (*Tinca tinca*) from Lake Bolsena (Latium region, central Italy). Scale bar = 100 μm. B) Metacercaria of *O. felineus* collected from a tench filet by digestion with 1% pepsin and 1% HCl. Scale bar = 100 μm. C) Egg detected in feces of the index patient of the August 2007 outbreak. Scale bar = 10 μm.

On August 4, 2007, 34 men from different villages in Viterbo Province attended a dinner in a private home, where they consumed marinated fish filets of tench (*Tinca tinca*) and of white fish (*Coregonus* sp.) from Lake Bolsena (Viterbo Province, central Italy). The fish had been frozen at –10°C for 3 days; they were then cut into filets ≈1-cm thick and marinated with vinegar and wine for 24 hours before consumption. On August 29, two of the men sought treatment at the hospital in Viterbo with symptoms of fever, abdominal pain, and diffuse myalgias; onset of symptoms had occurred 10 days earlier. In both men, laboratory findings showed marked leukocytosis (17.4 and 18.8 × 10^3^ cells/μL) with eosinophilia (10.1 and 13.9 × 10^3^ cells/μL) and elevated levels of alanine aminotransferase (ALT) (125 and 205 U/L). Examination of fecal samples showed *Opisthorchis* sp. eggs (Figure 1). Of the other 32 men who had attended the dinner at the private home, fecal samples of 18 were positive for *Opisthorchis* sp. eggs. Nine of these men had fever, nausea, abdominal pain, and myalgias. Specific IgG antibodies to *O. felineus* were detected only in the 20 men whose fecal samples were positive for eggs (attack rate 58.8%).

In all 11 symptomatic persons, onset of symptoms occurred ≈2 weeks after they consumed the fish. A patient with high levels of aspartate aminotransferase (AST) and ALT (315 and 899 U/L, respectively) was hospitalized for 7 days and completely recovered after 4 weeks.

All 20 infected persons showed eosinophilia (mean 4 × 10^3^cells/μL; range 0.27–14 × 10^3^ cells/μL), and 8 of them had elevated ALT levels (mean 182.6 U/L; range 57–899 U/L). Nine and 11 persons were treated with praziquantel (25 mg/kg orally 3×/day for 1 day) or albendazole (10 mg/kg/day orally in 2 doses for 7 days), respectively. In all 20 patients, there was complete remission of symptoms, and eosinophilia and aminotransferase levels returned to normal. Eggs were detected posttreatment in the fecal sample of only 1 patient who had been treated with albendazole for 7 days, although the patient’s leukocyte count was 6.9 × 10^3^ cells/μL and eosinophilia had disappeared. He was treated successfully with praziquantel.

On November 22, 2007, a woman sought treatment of fever and diarrhea at the hospital in Rieti. Laboratory investigations showed eosinophilia (19.6 × 10^3^ cells/μL) and elevated AST and ALT levels (118 and 364 U/L). Examination of a fecal sample showed *Opisthorchis* sp. eggs. The woman was treated with albendazole (400 mg/day orally in 2 doses for 7 days). Within 5 days, symptoms disappeared; aminotransferase levels slowly decreased, and the woman was discharged from the hospital. After treatment, no eggs were detected in the woman’s feces. She reported that 33 days before seeking treatment, she had eaten marinated tench filets at a restaurant. A friend of the woman was also present at the meal but he had only tasted the marinated tench filets. No eggs were detected in the fecal sample taken from the friend, although eosinophilia was slightly increased. Nonetheless, IgG antibodies to *O. felineus* were detected in a serum sample collected 58 days after the man had consumed tench (attack rate 100%). The restaurant owner stated that the origin of the infected fish was Lake Bolsena.

**Figure 2:**
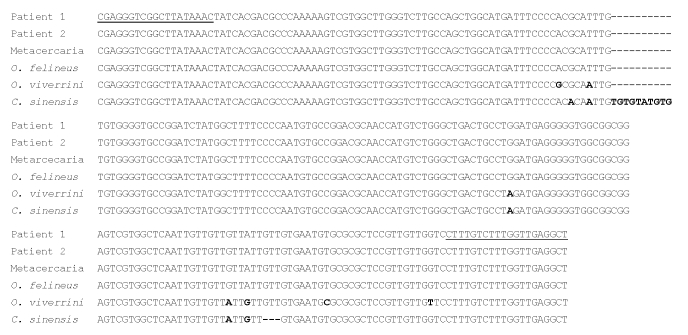
Alignment of the internal transcribed spacer 2 region of rDNA. Patient 1, DNA from eggs of a patient of the August 2007 outbreak; patient 2, DNA from eggs of the patient of the October–November 2007 outbreak; metacercaria collected from a tench (*Tinca tinca*) from Lake Bolsena (Latium region, central Italy); *Opisthorchis felineus*, reference DNA from an adult worm of *O. felineus* from Germany; *O. viverrini*, reference DNA from an adult worm of *O*. *viverrini* from Thailand; *C. sinensis*, DNA from an adult worm of *Clonorchis sinensis* from China. Primer sequences are underlined, different bases are in **boldface**, and gaps are represented by dashes.

Epidemiologic investigation of metacercariae in fish from Lake Bolsena showed a high level of infection in tenches (83.1% of the fish tested, range 1–146, median 4.5; Figure 1), yet metacercariae were not detected in any other species. To identify the species of *Opisthorchis*, we conducted PCR on eggs taken from patients’ fecal samples and on metacercariae from tenches. Eggs and metacercariae were identified as those of *O. felineus*; we observed no difference in the amplified sequences (GenBank accession no. EU926762) between eggs and metacercariae of Italy, and between parasites from Lake Bolsena and Germany (reference DNA) (Figure 2). In addition, we observed no difference in 16 of the 17 sequences deposited in GenBank (from metacercariae collected from the fish of several Russian rivers) with the exception of 1 (GenBank accession no. EF688142), which is different from the others because of the presence of an A instead of a T at position 32 of the ITS2 sequence (data not shown).

## Conclusions

In Italy, raw fish has become more popular in recent years, and the outbreaks we discuss reflect this change in eating habits. In fact, although *O. felineus* has apparently been circulating in Italy at least since the 19th century (*7*), the lack of cases of infection may be attributable to the low commercial value of the tench and the fact that it is traditional to cook fish well done in Italy.

In the 4 outbreaks in Italy, i.e., those investigated in 2003 and 2005 (*9*) and the 2 outbreaks that we investigated, the incubation period in symptomatic persons ranged from 2 to 4 weeks, which is consistent with reports in the literature (*12*). The attack rate was 100% in all but 1, the August 2007 outbreak (attack rate of 58.8%). This finding may be due to the fact that not all of the persons present at the private dinner had eaten marinated tench.

In the 4 outbreaks, 19 (59%) of the 32 infected persons were asymptomatic; no one had severe symptoms. The other 21 persons had only mild symptoms, probably because of the low number of parasites ingested and because infected persons did not regularly eat marinated tench. This epidemiologic and clinical picture differs from that observed in endemic regions of eastern Europe and Asia, where people frequently eat raw fish infected with *O. felineus* and where more severe symptoms have been reported (*12**,**13*).

Praziquantel and albendazole were effective treatments for all case-patients, except for 1 man, who had the greatest number of eggs in his stool sample. He was first treated unsuccessfully with albendazole and then successfully with praziquantel. Our data confirm the efficacy of praziquantel; however, albendazole is also apparently effective in eliminating worms and can be used when praziquantel is not available.

After the 2 most recent outbreaks, the local health service informed restaurant owners, fishermen, and the population in the areas of Lakes Bolsena and Trasimeno about the risks related to eating raw fish. At the same time, epidemiologic surveys on stray cats in the area showed that *O. felineus* eggs were present in their feces, with a prevalence of infection ranging from 23.5% to 40.0% (*14**,**15*). Given that most of these cats had eaten garbage from restaurants and fish carcasses discarded by fishermen, educating restaurant owners and fisherman on proper garbage disposal is important. According to the literature, metacercariae may be killed by freezing at –10°C for 5–70 days or at –28°C for 24 hours, depending on the size of the fish (*13*). In the August 2007 outbreak, the fish had been frozen at –10°C for 3 days, which did not completely kill the metacercariae. Even if fish are frozen in a home freezer, there is no way of knowing the internal temperature of the fish. Consumers should be warned about the risk of consuming raw fish regardless of where it has been frozen.
